# Infection control in delivery care units, Gujarat state, India: A needs assessment

**DOI:** 10.1186/1471-2393-11-37

**Published:** 2011-05-20

**Authors:** Rajesh Mehta, Dileep V Mavalankar, KV Ramani, Sheetal Sharma, Julia Hussein

**Affiliations:** 1 Department of Community Medicine, College of Medical Sciences, Bhavnagar, India; 2 Public Systems Group, Indian Institute of Management, Ahmedabad, India; 3 School of Health and Social Care, Bournemouth University, Bournemouth, England; 4 Immpact, University of Aberdeen, Aberdeen, UK

## Abstract

**Background:**

Increasingly, women in India attend health facilities for childbirth, partly due to incentives paid under government programs. Increased use of health facilities can alleviate the risks of infections contracted in unhygienic home deliveries, but poor infection control practices in labour and delivery units also cause puerperal sepsis and other infections of childbirth. A needs assessment was conducted to provide information on procedures and practices related to infection control in labour and delivery units in Gujarat state, India.

**Methods:**

Twenty health care facilities, including private and public primary health centres and referral hospitals, were sampled from two districts in Gujarat state, India. Three pre-tested tools for interviewing and for observation were used. Data collection was based on existing infection control guidelines for clean practices, clean equipment, clean environment and availability of diagnostics and treatment. The study was carried out from April to May 2009.

**Results:**

Seventy percent of respondents said that standard infection control procedures were followed, but a written procedure was only available in 5% of facilities. Alcohol rubs were not used for hand cleaning and surgical gloves were reused in over 70% of facilities, especially for vaginal examinations in the labour room. Most types of equipment and supplies were available but a third of facilities did not have wash basins with "hands-free" taps. Only 15% of facilities reported that wiping of surfaces was done immediately after each delivery in labour rooms. Blood culture services were available in 25% of facilities and antibiotics are widely given to women after normal delivery. A few facilities had data on infections and reported rates of 3% to 5%.

**Conclusions:**

This study of current infection control procedures and practices during labour and delivery in health facilities in Gujarat revealed a need for improved information systems, protocols and procedures, and for training and research. Simply incentivizing the behaviour of women to use health facilities for childbirth via government schemes may not guarantee safe delivery.

## Background

India accounts for nearly a fifth of all maternal deaths worldwide, so is an important target country in global efforts to reduce maternal mortality [1-3]. The current maternal mortality ratio in India is 230 per 100,000 live births [1], with World Health Organization [1], independent studies [2] and national reports [4] broadly concurring. Over the last decade, India intensified its efforts to improve maternal health and reduce maternal mortality. In 2000, the National Population Policy and Reproductive and Child Health Program II set out the goals for 2010 including: 80 percent of all deliveries in institutions; 100 percent of deliveries attended by trained personnel; and reduction of maternal mortality ratio to less than 100 per 100,000 live births. The National Rural Health Mission (2005-2012) aimed to improve availability of and access to maternal and child health services [5]. One of the main elements of the Mission was the continued promotion of institutional deliveries. Increased deliveries in health facilities have been achieved through financial incentives provided to health workers and to women. Institutional delivery rates have steadily increased in the last 13 years from 26% to 41% in 2005 [6].

Many wound and genital tract infections can be introduced during childbirth. Of these, puerperal sepsis is one of the most serious and life threatening. In India, maternal deaths from puerperal sepsis constitute the second most common cause after haemorrhage, accounting for approximately 15% of all maternal deaths [7]. It is recognised that some of the cases of haemorrhage also have infection as an underlying contributory factor [8]. There are considerable variations in estimates of the contribution of sepsis to maternal death. A sixteen year study from Northern India found that sepsis was responsible for over 35% of maternal deaths [9] and a study in Southern India revealed that sepsis was a leading cause of maternal death, responsible for 41.9% of deaths [10]. Demographic and health surveys show that the majority of women do not receive a postnatal check and 14% of women who had a birth in the last 5 years reported very high fever in the postpartum period [6]. There are few recent studies and little information on infections during childbirth.

Gujarat state performs comparatively well compared with the rest of the country on many maternal health indicators. Health centres, family welfare centres and general hospitals are widely found in urban areas, while the public health infrastructure in rural areas consists of primary health centres and community health centres. The state level maternal mortality was 160 in 2006 [4] and maternity care uptake was higher than the overall India average with over 50% institutional deliveries in 2005 [6]. Serious shortages of health professionals characterise especially the rural public health system. Much of the health infrastructure does not function due to these chronic staff shortages, and also because of the lack of drugs, supplies and equipment [11].

In Gujarat, puerperal sepsis was reported to cause almost 7% of maternal deaths, although the study found only small numbers of cases and concluded that underreporting was likely [3]. A number of specific factors made Gujarat state a suitable site for our study. The health sector provides an environment conducive to allow the conduct of research integrated within the health system. The Gujarat government and other key stakeholders in the state are supportive of a focus on infection control, and the work fits in with a state level initiative for a hospital accreditation scheme which requires specific criteria and standards to be met on various aspects of health service provision. Standards for infection control are not set in this scheme as yet, so the guidelines used to inform in this study was based on the principles of the World Health Organization's Global Patient Safety Challenge. This initiative on healthcare associated infections provides guidelines for clean practices, clean equipment, a clean environment and the availability of diagnostics and treatment [12-15].

The objective of this study was to assess infection control procedures and practices during childbirth in health facilities. The study was conducted as a preliminary needs assessment to inform the design of a subsequent intervention study to reduce infection rates in women who deliver in health facilities. The primary research question was: What are the current procedures and practices for infection control as reported by staff and through observation by researchers, in the labour and delivery areas in relation to:

• Management systems (health information data, protocols)

• Clean practices (hand washing, antisepsis, asepsis, surgical procedures)

• Clean equipment (gloves, gowns and instruments)

• Clean environment (surfaces, washing facilities)

• Diagnostics and treatment (blood products, antibiotics)

## Methods

The study was carried out from April to May 2009 in 20 health care facilities within two districts of Gujarat.

### District and health facility selection

Two districts were selected within Gujarat as 'better' and 'poorer' performing, based on reproductive and child health programme data indicators such as uptake of antenatal care, deliveries with health professionals, family planning and immunization. The better performing district was Ahmedabad and the other was Surendranagar. Twenty participating health facilities were selected to represent a range of care settings (Table 1). The facilities were chosen from those conducting deliveries, including: public, private for profit and private charitable facilities; and facilities at different levels of the health system, i.e. primary health centres, first referral and tertiary referral hospitals.

**Table 1:** Profile of health facilities included

Facility type	FRU*	Primary health centres	Private	Public (urban)	Tertiary	Private non profit	Total
Ahmedabad	2	2	1	3	1	1	10
Surendranagar	2	2	1	1	1	3	10
Total	4	4	2	4	2	4	20

* FRU = First Referral Unit, a health facility designated in India that provides hospital level obstetric services with inpatient beds and operation theatre facilities

The district health officer and the other officers in the selected facilities were informed of the study. Contact was made with the hospital supervisor and administrator to meet the officer in charge in selected facilities. The highest level of officer available at time of the visit was taken as the officer in charge of the facility. A preparatory meeting was held whereby the purpose of the study was explained to the officer in charge. Informed consent and permissions for the study were sought and a suitable time and day for the data collection agreed.

### Data collection

In each participating facility there were two modes of data collection:

a) a semi-structured interview with the officer in charge, and/or the health provider responsible for infection control procedures and practices.

b) a 'walk-through' of specified delivery care units to observe infection control procedures and practices. The rooms included in the walk-through were the labour wards, operation theatres, any obstetric emergency evaluation areas (such as an admission room), changing rooms, scrub rooms, autoclave rooms and instrument processing and storage areas.

Development of the data collection instruments was based on existing tools and guidelines for infection control [12-16]. Topic areas covered in the interview and observation tools were:

- General information: on the type of facility, caseloads, procedures and other materials related to infection control, such as registers, posters, documents or charts with summary information on infections.

- Practices: hand washing, use of non touch techniques and skin preparation.

- Equipment and supplies: use and methods for sterilisation of gloves, gowns and other instruments including availability and use of autoclaves, thermometers, disinfection practices and antiseptics.

- Environment: availability of running water, design of taps, sink placement and cleaning of contaminated surfaces.

- Diagnosis and treatment: availability, distribution and use of thermometers, bedside temperature recording charts, microbiological analysis and antibiotics.

Respondents were also asked to recall any cases of 'puerperal sepsis' which they had encountered. A definition of puerperal sepsis was not specified and the health worker freely allowed to describe the events they thought relevant.

Data collection was performed by five researchers experienced in community health, including the lead author (RM). They were not service providers in the participating districts. The data collectors were not blinded to the selection of districts. Training on data collection was conducted by RM. Informed consent was obtained from each of the respondents and officers in charge of the facility. All health care facilities were assigned with a unique identifier and analysis of data used only these identifiers. Ethical approval was sought from and granted by the Research and Publication Committee of the Indian Institute of Management, Ahmedabad. Permission for the study was also gained from the Health Commissioner of Gujarat.

### Analysis

Quantitative data was entered for each question. Single data entry was conducted using Epi info. Our analysis of quantitative data focused on the trends and norms of infection control procedures and practices specific to intrapartum care. Simple descriptive frequency measures were calculated. Qualitative data comprised narrations of interviewees' recollections of cases of puerperal sepsis.

## Results

Twenty health facilities (Table 1) participated and there were twenty respondents, one from each facility. There were no refusals. Most of the respondents were medical doctors (40% medical officers and 30% obstetricians) and 25% were nurses or nurse midwives. One respondent was the officer in charge of the operation theatre. Almost all respondents had at least two years experience in that particular health facility.

All the facilities conducted deliveries. Obstetricians, doctors, nurses or midwives were responsible for conducting deliveries in the facilities. In the preceding year, the primary health centres conducted between 5 and 77 deliveries, intermediate health facilities (private and public) between 450 and 600 deliveries, first referral units (health facilities providing government designated obstetric services) 550 to 840 deliveries and tertiary hospitals 800 to 1800 deliveries. More than 400 deliveries were carried out annually in 60% of facilities. Half of the facilities had more than 20 beds, although 10% did not have designated maternity beds. A separate antenatal ward was available in 70% of the facilities and a separate postnatal ward in 20%. Ninety percent of facilities had a separate operation theatre and labour room. Two facilities (10%) had a combined labour room and operation theatre (in data analysis this space was considered as a labour room).

Infection control procedures and practices reported during interviews are summarised in Table 2, and those observed are in Tables 3 and 4. The findings from the two different data collection activities are presented together in the narrative below.

**Table 2:** Infection control procedures and practices, as reported during interviews

	% (N = 20)
A standard procedure exists	70

*Type of information available*	
Book and chart showing infection rates	5
Chart only	15
Written procedure available	5
Verbal procedure reported	45

*Management/procedural activities conducted*	
Infection control committee (monthly meetings held)	15
Case(s) of hospital acquired infection recorded	5
Audit or maternal death review	10
Any staff member attended training in infection control in last year	25

*Details of clean practices or asepsis*	
All staff routinely wash hands before procedures.	95
Soap available at all times for hand-washing.	95
Staff vigorously rub hands together with antiseptic or soap and water before any aseptic procedure such as a vaginal examination during labour.	75
Sterile gloves	80
Patients are advised for prevention of infection	95

*Diagnosis and treatment*	
Blood culture can be taken in facility	25
Staff aware of common organisms found in blood/pus/fluid culture reports	20
Antibiotics available in facilities for organisms found	95

**Table 3:** Infection control procedures and practices observed in general maternity areas

	% (N = 20)
Wall posters and charts relevant to infection control	35
Thermometer available on the ward	65
Patients charts with temperature recorded regularly	45
Soap (or any other antiseptic) available	90
Antibiotics seen	90

### Management and procedural activities

Seventy percent of respondents said that standard infection control procedures were being followed in their facility although they indicated that procedures in written form were lacking (Table 2). A total of 25% of respondents indicated that books, charts or some form of written procedure containing information on infection rates or infection control procedures were available. Wall posters and charts relevant to infection control were actually observed on display in 35% of facilities (Table 3). Most were faded and old, did not contain current data, were few in number (one or two in each facility) and/or were not present in prominent places.

Most facilities did not keep systematic data on infection rates in the maternity units. Delivery registers were seen during observation. The registers contained information about delivery date and time, sex and birth weight of newborn and type of delivery, although details pertaining to indicators of infection and other crucial information for data analysis of clinical conditions was lacking. Where data was available, infection rates were found to be between 3% and 5%. Respondents also described a number of activities that identified problems with, or created awareness of infection control during childbirth. These included meetings of infection control committees, maternal death reviews, audits, training and feedback on infection rates. These activities were conducted only in the minority of health facilities (Table 2). Staff commonly complained that their heavy workloads made it difficult to follow infection control practices. Most health personnel indicated that they would like to see improvements in the standards for infection control.

### Clean practices and asepsis

In almost all health care facilities, the respondents reported that they and their staff routinely washed their hands before and after procedures, although only 75% of respondents reported vigorous rubbing of hands before conducting what were supposed to be aseptic procedures (Table 2). Eighty percent of respondents believed that the frequency of hand washing in their facility was "good", while the rest believed their practices were "average". Aseptic practices were also thought to be good with 70 to 100% of respondents saying for example, that surgical sites were prepared from the centre outwards with aseptic solutions; that if there was a break in the sterile field (such as a hole in the glove) this was identified and the sterile field re-established; and that in-patient pregnant women were given advice for the prevention of infection.

Soap and sterile gloves were reported as being widely available in 80% or more of facilities. Surgical gloves were washed and prepared for reuse in more that 70% of facilities, reportedly to limit costs. It was also observed that new sterile gloves were not available in the labour room. Staff present during the observation exercise said that vaginal examinations in the labour room were conducted with reused, washed or autoclaved gloves. Sterile gloves were generally reserved for the operation theatre. For vaginal examination in some places, gloves were used after simple washing without autoclaving. Alcohol rubs were not used at all and not seen during the observation exercise. Running water was available in labour rooms and operation theatres in almost all facilities, although a third of facilities were observed not to have wash basins with "hands-free" taps (Table 4).

**Table 4:** Observations across different areas of health facility

Items available	Labor room,% (N = 20)	Operation theatre, % (N = 18)
24-hour running water	95	100
Wash basin with elbow or knee tap	65	61
Soap	90	80
Antiseptics for skin preparation	95	94
Sterile (unused) gloves	0	89
Surgical gloves: reused	85	71
Sterile linen packs	65	66
Sterile delivery packs	50	0
Disposable delivery kit ("Mamta kit")	40	0

### Clean equipment and supplies

In general, reported and observed availability of equipment and supplies agreed. Protective clothing such as aprons, gloves, caps and face masks were available in over 80% of facilities, although some items such as nail brushes were present in only about 5% of facilities. Sterile gowns, linen packs, delivery packs and packs for Caesarean section were found in only 40-65% of facilities (Table 4). Sterile disposable delivery kits (Mamta Kit) which are supplied for home deliveries, were found in 40% of the facilities. Autoclave machines were available in most facilities, but indicator paper only in 65% of the facilities. A register for recording of autoclaving was maintained in most facilities.

### Clean environment

Respondents reported that the floors of the labour room and operation theatre were wiped once a day in a quarter of the facilities, twice a day in 25% of facilities, and three times a day in 45% of the facilities. One facility reported wiping the floors four times a day. Regular maintenance of checklists related to infection control procedures was observed only occasionally.

Fluids used for wiping floors were Phenol, Dettol, Lysol and chlorine powder. Respondents indicated that in general, the cleaning of the operation theatre was given more importance (done after each case is completed) than in the labour rooms where cleaning was variable. Only 15% of facilities reported that wiping of surfaces was done after each delivery in the labour rooms. Various mechanisms were used to disinfect operation theatres, including fumigation with a formalin-potassium permanganate combination, spirit sprays, ultraviolet air purification and manually wiping walls, furniture and floors.

### Diagnosis and treatment

Thermometers were only available in 65% of facilities. Evidence that recording of temperatures was monitored (by bedside charts, clinical notes) was found in only 45% of facilities (Table 3). Staff reported that blood cultures could be taken in only 25% facilities. In one facility, the interview revealed that swabs were collected for culture from different areas in the operation theatres on a monthly basis.

Respondents indicated that antibiotics were freely available at the majority of facilities. Antibiotics were observed to be available on the wards. Researchers were told that antibiotics were given to most of the women undergoing (even normal) delivery by the oral route for 14 days or as a single intramuscular injection. A few facilities (15%) reported occasional difficulty in procuring antibiotics.

### Experience of recent cases of puerperal sepsis

At most facilities, respondents could not recall a recent case of puerperal sepsis. In two facilities, two doctors recalled one case each.

Case 1*: "....2 years back. The infection was acquired outside *[the facility]. *Patient *[was] *admitted in poor general condition*. [She was] *unable to take food*. [The patient] *delivered at home in the hands of an untrained person...was diagnosed on the basis of the presenting complaint, blood counts and home delivery. Treatment was parenteral antibiotics in fluids and symptomatic treatment...recovered within two days."*

Case 2: "*A case of puerperal sepsis...five years *[ago]. [The patient] *came in morning to *[out patients] *for follow up and she was found to have foul smelling discharge. With further examination it was found to be puerperal sepsis and the cause identified was lack of removal of a gauze piece from the vagina. Nursing staff forgot to remove it*. [From] *then onwards it was decided to do vaginal examinations before discharge of any patient*. [Patient] *was treated with injection ceftriaxone, metronidazol, local vaginal wash with Betadine..."*

During the observational exercise however, informal talks with different categories of health care staff indicated that infections in maternity wards were observed more commonly than openly discussed.

## Discussion

The National Rural Health Mission's investment in a scaled-up drive to encourage institutional delivery stipulates attention to quality of care during childbirth in health facilities. The high uptake of maternity care services in Gujarat highlights the importance of improving quality of care in health facilities. One of the markers of quality care is the prevention and treatment of infections, so attention to infection control during provision of maternity services is paramount in order to achieve India's desired reductions in maternal mortality. Our study shows that infection control is likely to be suboptimal in many delivery units in Gujarat. There is lack of a systemic approach to infection control in facilities with no set procedures for recording, analysis or follow up action. This is evidenced by the lack of standard guidelines and infection control committees, and poor data availability, feedback and audit in the majority of facilities, even in a relatively well performing state like Gujarat.

Individual awareness and practices of hand washing and asepsis appear to be established to some degree. The availability of equipment, basic supplies and antibiotics is good but not universal. Basic environmental, protective and sanitary conditions in the health facilities are not ideal and cleaning procedures ad hoc and disorganized. Diagnostic facilities are poor. Antibiotics are widely and irrationally used. There are some indications that sterilization and cleanliness of operating theatres is better than in labour rooms - for example, unused gloves are reserved for theatre use (Table 4) and practices for cleaning of surfaces superior.

Data on infection rates was hard to find, so we asked health workers to recollect cases as a rough gauge of whether serious infections after childbirth were commonly encountered. Puerperal sepsis is usually defined clinically, with fever, uterine involvement and bloodstream infection occurring after childbirth, although definitions vary [17,18]. The cases narrated were not clearly confirmed as puerperal sepsis but could have been infections related to childbirth. The apparent rarity of such events could be explained in various ways - recall bias (not remembering cases), underreporting, successful infection control, wide use of antibiotics preventing infections or rapid and effective treatment of early signs and symptoms. With poor recording systems for infections, underreporting is likely. The liberal use of antibiotics may be preventing infections, but such practices raise other issues of cost, evidence use in clinical practice and antibiotic resistance.

This needs assessment has a number of limitations. The data collectors were not blinded to how the facilities were selected, so they may have recorded findings in a biased way, especially between facilities in the less and better performing districts. The prevention of infections in hospitals is a sensitive issue as it pertains to individual behaviours and practices, so there may have been fears of reprisals in health facility staff, affecting their responses in spite of assurances that the researchers provided during data collection. Health facilities were first informed that the study was on infection control. The observation visit was organised in advance and by appointment on a specific day, so changes could have been made to specially prepare for the visit. The interview and observation data collection tools were formulated to complement each other, rather than to cross check the findings. Comparison between the two is therefore limited, although there appeared to be broad agreement in some indicators such as the low availability of written infection control procedures and availability of gloves, soap and antibiotics.

## Conclusion

Based on the guidelines and actions described in the Global Patient Safety Alliance framework and other infection control standards [12-15,19], key recommendations proposed are as follows:

• Given the lack of information, underreporting of puerperal sepsis and other infectious complications relating to childbirth is likely. Record keeping, analysis and feedback of data needs to be improved. Criteria for diagnosis of puerperal sepsis should be uniformly laid down and communicated. Notification of puerperal sepsis should be encouraged.

• Protocols for infection control measures should be prepared, standardised and adapted to local situations. The protocols should include assessment of the evidence for procedures like fumigation, reuse of items, maintaining cleanliness of the environment (especially in labour rooms) and rational use of antibiotics, adapted to resource constrained settings.

• Training to upgrade minimum skills for infection prevention should be started at pre-service level and extend to continuous training at service for staff. This should be based on assessments of staff needs. The means to create motivation and accountability at individual level should be explored.

• Community based research should be carried out to estimate the burden of infection in postnatal mothers. Factors associated with occurrence of infection should be documented. This needs assessment can provide the basis for such work.

• There should be active hospital infection control committees set up, combined with maternal death reviews, audits, training and feedback on infection rates. State level officers could be included in such activities to ensure integration of these activities within the health system as a whole. Their role should also be to ensure the link-up between increasing utilization (for example, through incentives) and improving the quality of care that women receive once they reach health facilities.

Although the increasing institutional delivery rates in Gujarat is likely to benefit the safety of mothers and babies overall, there is need for 'watchfulness' in the light of the transition to facility based childbirth. Studies from several countries have demonstrated that mothers who had planned home deliveries had fewer infections than those who delivered in hospitals [20,21], although it should be stressed that the settings, systems and home environments are important contextual factors that can change the balance of effects. Incentivizing health uptake behavior has the risk of causing adverse clinical outcomes unless health care is concurrently enhanced. We conclude that contracting infections during childbirth in health facilities is a risk in Gujarat and India that is poorly documented. A focus on infection control during delivery and puerperal sepsis may help to improve quality of maternity care. Models of care which try to drive uptake of health services need to be evaluated not only in terms of increased utilization, but also from the perspective of the quality of care provided.

## Competing interests

JH is funded by the University of Aberdeen, DVM and KVR by the Indian Institute of Management, Ahmedabad. They receive part support of their salaries from the MacArthur Foundation through this grant. The project work conducted by RM and SS was funded by the MacArthur Foundation grant. Material from this paper was used to apply for a subsequent grant. The funders and employers of the authors have no responsibility for the information provided or the views expressed in this paper. The views expressed herein are solely those of the authors.

## Authors' contributions

RM conducted the study, performed analyses and wrote the report upon which this paper was based. DM and KVR contributed to study design and analysis. SS conducted literature searches, developed the tools and participated in the field work. JH conceived the study and drafted the manuscript. All authors read and approved the final manuscript.

## Pre-publication history

The pre-publication history for this paper can be accessed here:

http://www.biomedcentral.com/1471-2393/11/37/prepub
